# The Emotional Characteristics of Schizotypy

**DOI:** 10.4306/pi.2008.5.3.148

**Published:** 2008-09-30

**Authors:** Seon-Ah Yoon, Do-Hyung Kang, Jun Soo Kwon

**Affiliations:** 1 Neuroscience Institute, SNU-MRC & BK21 Life Sciences, Seoul National University, Seoul, Korea.; 2 Department of Psychiatry, Seoul National University College of Medicine, Seoul, Korea.

**Keywords:** Schizotypy, Psychosis, Risk factor, Emotion, Characteristics

## Abstract

**Objective:**

The aim of this study was to investigate the relationship between emotional traits and schizotypal symptoms and to establish a hypothetical model for the causal relationship between them.

**Methods:**

Schizotypal symptoms were assessed using the Schizotypal Personality Questionnaire (SPQ), and a total of seven emotional traits considered to be potential risk factors for schizotypy were categorized as emotional disturbances, emotional attenuators or emotional amplifiers. A total of 502 undergraduate students completed the SPQ and other scales.

**Results:**

The result of the present study suggested that the high levels of emotional disturbance in individuals who are prone to schizotypy or psychosis are amplified by their intensity and fluctuation. However, if their emotion attenuating abilities function well, these disturbances can be controlled and the schizotypal symptoms and progression to psychosis can be contained. Discriminant analysis showed that 69.0% of the subjects with many schizotypal symptoms and 80.7% of the subjects with few schizotypal symptoms were correctly classified.

**Conclusion:**

The present study suggests the possibility of using emotional traits to identify the risk factors for psychosis.

## Introduction

Schizotypy refers to the phenomenon of experiencing 'psychotic' symptoms^1^ and may be conceptualized as a predisposition to schizophrenia.^2^,^3^ Schizotypy usually indicates a 'liability' to schizophrenia, or more generally to nonspecific 'psychosis proneness'.^4^ Thus, it is important to identify the risk factors for schizotypy because early identification may enhance the etiological understanding of schizophrenia spectrum disorders and facilitate the development of more efficient prevention and remedial treatments.^5^

Previous studies performed thus far have provided a solid foundation for understanding the symptoms and emotional states of individuals with schizotypy. However, the previous studies had several limitations. The first problem is related to the importance of the study of schizotypy or psychosis proneness in a general population. A growing number of studies consider psychosis as a continuum with normal functioning at one end and abnormal functioning at the other end.^1^,^6^,^7^ Thus, exploring the risk factors modulating the expression of schizotypal signs in non-clinical populations beyond the clinical concept of schizophrenia would better contribute to the elucidation of the etiology of psychosis than research restricted to subjects at the endpoint of the distribution of the psychotic dimension.^7^ Second, despite the importance of identifying risk factors, various emotional traits were not examined in an integrated manner. Since emotional disturbances, such as flattened and inappropriate affect, are regarded as characteristic of schizophrenia,^8^ emotion has been an important issue in the study of psychosis. A previous study indicated that one of the cardinal dysfunctions associated with schizophrenia concerns the processing of emotional information, including disturbances in the expression, experience and perception of emotions.^9^ However, in many studies, only single emotional traits, such as depression or anxiety, were examined, and other emotional traits had not been studied extensively at all.^10^,^11^ Even though many emotional traits can aid in the understanding of the onset and progress of psychosis when applied to mental disease, extensive investigation of these variables has not been carried out, and there is no appropriate frame in which to integrate the variables.

Considering the previous studies and their limitations, in this study we tried to investigate the relationships between emotional traits and schizotypal symptoms and to establish a hypothetical model of their causal relationship. To categorize the rest of the emotional traits, we introduced the concept of emotional attenuators and amplifiers, which process personal emotion through opponent functions. That is, while emotional amplifiers are likely to enhance negative affect, complementary emotional attenuators seem to reduce the emotional extremes to manageable levels. The terms "emotional amplifiers" and "emotional attenuators" were originally proposed by Tomkins,^12^ and similar concepts, such as affect control and affect infusion or mood incongruent and mood congruent personality variables were suggested by other emotion researchers.^13^,^14^ The emotional attenuators used in this study included mood awareness and repair, which are considered to be emotional traits that control and regulate one's emotional expressions in order to calibrate and maintain emotional equilibrium.^14^-^16^ In contrast, the emotional amplifiers used in this study included mood reactivity and affect intensity, which are considered to be emotional traits that generate and intensify negative affect.^15^,^17^

It is hypothesized that emotional disturbances and emotional amplifiers will be positively correlated with schizotypal symptoms, whereas emotional attenuators will be negatively correlated. In addition, when emotional disturbances affect schizotypal symptoms, it is hypothesized that emotional attenuators and amplifiers assume the roles of intermediate variables and exert negative and positive influences, respectively. That is, emotional amplifiers will magnify the negative emotion to increase the schizotypal symptoms, and emotional attenuators will control the negative emotion in order to reduce it.

## Methods

### Participants

A total of 502 undergraduate students (233 male and 269 female; mean age 20.33 years±2.34) completed the Schizotypal Personality Questionnaire (SPQ)^18^ and other scales measuring emotional disturbances, emotional attenuators and amplifiers. They were enrolled in undergraduate introductory psychology classes at four different universities in Seoul and Gyeonggi province, and they participated in the study to obtain course credit. Data from five students were discarded because their questionnaires were not completely filled out. The test results of emotional traits were sent to all of the participants in order to improve their motivation to participate and thereby ensure quality results. With the informed consent of the subjects, the assessment tools were administered.

### Instruments

#### Measure of Schizotypy

The SPQ^18^,^19^ is a self-report questionnaire comprised of 74 true-false questions that assess cognitive, perceptual, affective, and interpersonal features consistent with the symptoms of schizotypal personality disorder (SPD) as defined by the Diagnostic and Statistical Manual of Mental Disorders Third Edition (DSM-III-R).^20^ The SPQ generates nine separate scores, one for each of the nine DSM-III-R SPD criteria. It can be used as a screening tool in the general population for the identification of risk factors for susceptibility to psychosis, and it may serve as a measure of individual differences in schizotypal personality and as an indicator of the biological-genetic vulnerability to schizophrenia.^21^

The internal consistency of the total SPQ score was 0.91, while the alpha values for individual subscales ranged from 0.71 to 0.78, with a mean of 0.74. The total alpha score for the Korean version of the SPQ is 0.91, and that of the individual subscales ranges from 0.73 to 0.83, with a mean of 0.77. The SPQ has high test-retest reliability (r=0.82). Deviance on the SPQ has been shown to identify schizotypal personality disorder such that 55% of individuals scoring within the top 10% of total SPQ scores met DSM-III-R criteria for schizotypal personality disorder.^22^

#### Measures of Emotional Disturbances

The measures of emotional disturbances experienced by the subjects consisted of three important negative emotions: depression, anxiety and anger. The depression, anxiety and anger-hostility subscales of the Symptom Checklist 90-Revised (SCL-90-R)^23^,^24^ were used to measure these traits. The SCL-90-R consists of ratings of 90 symptoms on a five-point scale (0=not at all to 4=extremely) indicating how frequently the client has experienced these symptoms in the last week. It is usually scored on nine primary symptom dimensions, and it helps evaluate a broad range of psychological problems and symptoms.

Internal reliabilities have been reported to be 0.92, 0.88 and 0.73 (Korean version: 0.89, 0.86 and 0.68) for depression, anxiety and anger, respectively.

#### Measures of Emotional Attenuators

Clarity of feelings and mood repair subscales of the Trait Meta-Mood Scale (TMMS)^25^,^26^ were used to measure the ability to perceive and regulate one's own emotions. The TMMS includes three subscales, and it is a 30-item self-report measure to which participants respond on a five-point scale (1=strongly disagree to 5=strongly agree). Internal reliabilities of 0.88 and 0.82 (Korean version: 0.84 and 0.72) have been reported for clarity of feelings and mood repair, respectively.

#### Measures of Emotional Amplifiers

Emotional amplifiers were measured using the mood reactivity subscale of the Mood Survey for emotional fluctuation^27^,^28^ and the Affect Intensity Measure (AIM)^29^,^30^ for emotional intensity. The Mood Survey was developed to describe the general levels of mood, frequency of mood changes and intensity of reaction to mood-altering events. The questionnaire consisted of 15 items on a six-point scale ranging from "strongly agree" to "strongly disagree", with two subscales.

The AIM is a widely used measure of stable individual differences in the typical magnitude of an individual's experience of emotion. It consists of 40 items, with six-point scales indicating how often the person reacts and feels the way the items indicate. The internal reliability of the AIM has been reported to be .091 (Korean version: 0.87).

### Statistical analysis

For all of the research scales used in this study, correlations between variables, Cronbach alpha reliabilities, and scale means were determined. Correlations between the variables were calculated using Pearson's correlation coefficients. A two-tailed probability of less than 0.01 was considered statistically significant. Structural equation modeling (SEM) was conducted to verify whether the emotional traits affect schizotypal symptoms. Emotional disturbances (depression, anxiety and anger), emotional attenuators (mood clarity and mood repair), emotional amplifiers (mood reactivity and affect intensity) and SPQ score were included in order to examine the hypothesized model. A two-tailed probability of less than 0.001 was considered statistically significant. In addition, all of the variables used in SEM were included in the discriminant analysis, and independent sample t-tests were conducted to examine the possibility that persons with many schizotypal symptoms can be distinguished from those with few schizotypal symptoms.

## Results

### Intercorrelations and general descriptions of the scales used in this study

Table 1 shows the intercorrelations, Cronbach alpha reliability coefficients, and mean scores of the emotional traits as well as the SPQ total scores of the sample population.

Analysis of the emotional disturbances showed that depression [r(502)=0.514, p=0.000], anxiety [r(502)=0.493, p=0.000] and anger [r(502)=0.464, p=0.000] had positive correlations with the SPQ total score. Analysis of the emotional attenuators revealed that mood clarity [r(502)=-0.469, p=0.000] and mood repair [r (502)=-0.297, p=0.000] had negative correlations with the SPQ total score. Analysis of the emotional amplifiers showed that mood reactivity [r(502)=0.444, p=0.000] and affect intensity [r(502)=0.255, p=0.000] had positive correlations with the SPQ total score.

The average Cronbach alpha coefficient of the emotional traits was 0.815, and the alpha value of the SPQ total score was 0.905.

### Structural equation model for the influence of emotional traits on schizotypal symptoms

When emotional disturbances influence schizotypal symptoms, it is hypothesized that emotional attenuators and emotional amplifiers assume the roles of intermediate variables and exert negative and positive influences, respectively. Structural equation modeling (SEM) was carried out in order to verify whether emotional traits do indeed have such an effect on schizotypal symptoms.

**FIGURE 1:**
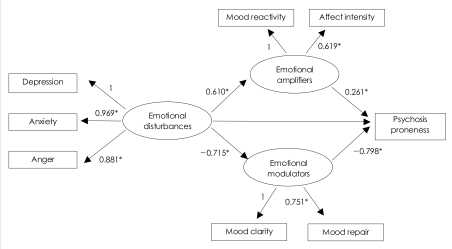
Structural equation model for the influence of emotional traits on schizotypal symptoms (n=502). ^*^p<.001. The numbers indicate regression estimate.

The result of the analysis of the total sample population is presented in Figure 1. The model was constructed so that emotional disturbances, such as depression, anxiety and anger, influenced schizotypal symptoms, as measured by the SPQ. In addition, the emotional attenuators, including mood clarity and mood repair, and the emotional amplifiers, including mood reactivity and affect intensity, would act as intermediate variables.

The indices of the model fit were fair [χ^2^(17)=111.447, p=0.000, RMSEA=0.105, RMR=0.058, GFI=0.946, NFI=0.916, CFI=0.927, AIC=149.447, R^2^=0.437]. The subscales of the emotional traits included in the structural equation model had statistically significant path coefficient estimates. The emotional disturbances had a negative effect on the emotional attenuators (β=-0.715, p<0.001) and a positive effect on the emotional amplifiers (β=0.610, p<0.001). Emotional attenuators had a negative effect on the intermediate variables (β=-0.798, p<0.001), while the emotional amplifiers had a positive effect on schizotypal symptoms (β=0.261, p<0.001).

To validate this model, alternative models were hypothesized and compared with this model. First, the alternative model, which used only emotional disturbances and did not include emotional attenuators and emotional amplifiers, had good model fit [χ^2^(2)=1.677, p=0.432, RMR=0.010, RMSEA=0.000, GFI=0.998, NFI=0.998, CFI=1.00, AIC=17.677], but the explained variance (R^2^=0.352) was lesser than the model hypothesized in this study. The second model in which the direct path from emotional disturbances to schizotypal symptoms was added to the model of this study was examined for whether the additional path had significant influence. The model fit was similar to the model of this study [χ^2^(16)=111.342, p=0.000, RMR=0.058, RMSEA=0.109, GFI=0.946, NFI=0.916, CFI=0.926, AIC=151.342, R^2^=0.452]. However, even with one path added, the chi-square value was not efficiently reduced. In this case, when one path was added, the Chi value should be reduced to lower than 3.84, but only 0.15 Chi value was reduced. Finally, in the third model, emotional disturbances, emotional attenuators and emotional amplifiers predicted schizotypal symptoms separately, regardless of their interrelationship. The fit of this model was not good, and the explained variance was lesser than that of the model proposed in this study [χ^2^(18)=444.683, p=0.000, RMR=0.268, RMSEA=0.218, GFI=0.820, NFI=0.663, CFI=0.670, AIC=480.683, R^2^=0.371]. Therefore, the model that we suggested in this study was the most appropriate among the possible hypothesized models, considering both the explained variance and model fit.

### Discriminating individuals with many schizotypal symptoms using emotional traits

Discriminant analysis was used to examine the possibility that individuals with many schizotypal symptoms can be distinguished from those with few schizotypal symptoms using the emotional traits resulting from the structural equation model.

In a previous report investigating the criterion validity of the SPQ scores, six individuals (5.5% of data) were subjected to the Structured Clinical Interview for DSM-III-R Personality Disorders (SCID-II)^31^ and diagnosed with schizotypal personality disorder according to DSM-III-R criteria.^18^ The author calculated a conservative base-rate estimate for this disorder in the sample at 5.5% (55% of those scoring in the top 10% of the SPQ scores had a schizotypal diagnosis). Since high scorers on the SPQ are likely to result in a clinical diagnosis of schizotypal personality disorder, these data also suggest that the SPQ would be useful in research focused on schizotypal personality disorder. The cut-off point for the upper 5.5% of data was 41 in one study^18^ and 39 in another study.^32^ In the latter study, the cut-off point corresponding to the upper 5.5% of the data (29 persons) was 40, and this result was consistent with those of the preceding studies.

The variables entered in the analysis were selected from the SEM result and included depression, anxiety and anger as emotional disturbances, mood clarity and mood repair as emotional attenuators and mood reactivity and affect intensity as emotional amplifiers. For all of the emotional traits, the t-test showed that the subjects with many schizotypal symptoms had significantly higher scores than those with few schizotypal symptoms. Discriminant analysis revealed that 69.0% of the subjects with many schizotypal symptoms and 80.7% of the subjects with few schizotypal symptoms were correctly classified. The correct classification of cases was 80.0%.

## Discussion

The aim of this study was to investigate the influence of various emotional traits on schizotypal symptoms and to establish a possible hypothetical model for a relationship between them.

Among the efforts to identify the risk factors for schizophrenia, the presence of emotional disturbance has received great attention.^33^ The most frequent early signs of developing schizophrenia were emotional disturbances, such as depression, anxiety and restlessness.^34^ These symptoms were reported by 12-22% of schizophrenia patients, when they were directly asked about "the first thing they noticed that indicated that there was something wrong".^35^

Compared to studies of the association between emotional disturbances and psychosis, few studies have investigated the relationship between other emotional traits and psychosis. In one study designed to examine whether emotional traits are associated with schizotypy, schizotypal individuals reported greater intensity and attention to emotions, but less emotional clarity.^36^ Furthermore, in a study designed to investigate the relationship between personality disorders and emotional variables, the result indicated that individuals with Cluster A personality disorders, including schizotypy, lack emotional understanding (i.e., emotional clarity) and the ability to effectively regulate their emotional states (i.e., emotional repair).^37^

In the correlation analysis, emotional disturbances showed strong positive correlations with the SPQ score. While emotional disturbances and emotional amplifiers showed a positive correlation with the SPQ total score, mood clarity and mood repair as emotional attenuators showed a negative correlation with the SPQ total score, as we predicted. This result was consistent with the findings of previous studies, as schizotypal symptoms are positively correlated with affect intensity and negatively correlated with mood clarity and repair.^36^,^37^ It is generally acknowledged that emotional disturbances, such as anxiety, depression and irritability, can precede and accompany psychotic symptoms.^33^,^38^-^41^ Our results were consistent with those of previous studies.

The influence of emotional disturbances on schizotypal symptoms and the ability of emotional attenuators and emotional amplifiers to act as intermediate variables, decreasing or increasing the influence of emotional disturbances on psychosis, were investigated using the structural equation model. The results showed that the high levels of emotional disturbance in people who are prone to schizotypy or psychosis are amplified by their intensity and fluctuation. However, if their emotion attenuating abilities function well, these disturbances can be controlled and the schizotypal symptoms and progression to psychosis can be contained.

The results of this study have important preventative and therapeutic implications. A previous study showed that the "Training of Emotional Intelligence (TEI)" for schizophrenia has no treatment-specific effects.^42^ This result may be due to the lack of a theoretical background for emotional processing in schizophrenia. Indeed, a more effective therapeutic program can be devised if we can clearly understand the effect of emotional attenuators and amplifiers on psychotic symptoms.

Discriminant analysis showed that 69.0% of the subjects with many schizotypal symptoms and 80.7% of the subjects with few schizotypal symptoms were correctly classified. The correct classification of cases was 80.0%. Though there are some limitations to the present study, the results suggest the possibility that emotional traits can be used to identify risk factors for psychosis. Two research groups had proposed a range of theoretical models, depending on which affective mechanism leads to the psychotic symptoms.^43^,^44^ However, the former emphasized cognitive aspects and hallucination, while the latter focused on the role of stress in daily life. To the best of our knowledge, the present study is the first to try to establish an emotional pathway to psychosis through an extensive and integrative investigation of various emotional concepts.

The present study has several limitations, and several important research issues remain open to empirical investigation. First, because the dependent variable is a measure of "proneness to psychosis" in a supposedly normal population, it is difficult to know what proportion of these individuals would eventually convert to psychosis and what the distribution of proneness scores in a normal population looks like when compared to a population that would be considered more susceptible to the development of psychosis (i.e. the Ultra High Risk group). Therefore, both clinical and non-clinical samples should be included to allow for direct examination of the stability of any relationship across the continuum of psychopathology. Second, because the only measure of anything close to psychosis is a schizotypy questionnaire administered to undergraduates, other measures of psychosis development need to be applied in future studies. Furthermore, while structural equation modeling is informative, it remains limited by the fact that these data are cross-sectional and any predictive model must be confirmed by a longitudinal prospective analysis.

## Figures and Tables

**TABLE 1:**
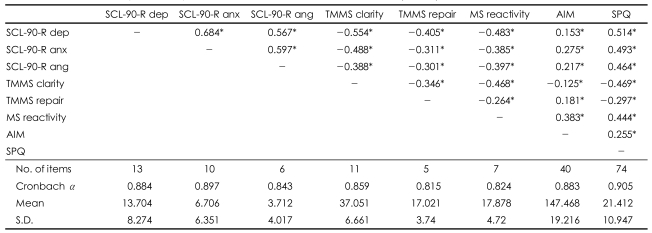
Intercorrelations, reliabilities, and means of emotional traits and SPQ score (N=502)

^*^p<.01. SCL-90-R dep: Depression subscale of Symptom Checklist 90-Revised, SCL-90-R anx: Anxiety subscale of Symptom Checklist 90- Revised, SCL-90-R ang: Anger subscale of Symptom Checklist 90-Revised, TMMS clarity: Clarity of feeling subscale of Trait Meta-Mood Scale, TMMS repair: Mood Repair subscale of Trait Meat-Mood Scale, MS reactivity: Mood Reactivity subscale of Mood Survey, AIM: Affect Intensity Measure, SPQ: Schizotypal Per sonality Questionnaire
